# Parliament and the Profession

**Published:** 1917-08-04

**Authors:** 


### Parliament and the Profession.

### Ministry of Health.

Mr. Bonar Law stated that it is not intended to introduce the proposed Bill for the Ministry of Health before the House of Commons rises for the recess.

### War Office Contracts with Medical Officers.

The Financial Secretary to the War Office informed Mr. Watt that medical officers whose contracts are for one year are allowed to return home on the expiration of that period, unless they elect to renew in the area of operations where they are serving.

### The Appeal for Shell-Shock Sufferers.

Mr. Barnes, Minister of Pensions, stated that an appeal, on behalf of recuperative hostels for sailors and soldiers invalided with nerve strain, referred to in a question by Mr. Stewart, had not been seen by Sir Frederick Milner, whose signature it purports to bear, and that the terms of the appeal do not accurately represent the facts with regard to these men. A special medical board has been empowered to deal with all such cases and to award pensions or gratuities to them. Cases invalided out of the Services before the board was set up are entitled to be re-examined, and local War Pensions Committees have been asked to report cases which, in their opinion, have not been suitably treated. All cases are entitled to treatment and training, and provision for this purpose has been made at an institution at Golders Green, which has 105 beds and will shortly open another 50. There is another institution at Belfast, and accommodation is being sought elsewhere. Each man is awarded a pension or a gratuity according to the circumstances of his case. The Ministry of Pensions hopes to carry out all the objects of the appeal to private charity referred to in the question.

### Unutilised Skill of Medical Men.

In a question to the Under-Secretary of State for War, Mr. Watt called attention to the use of eminent medical men, with special qualifications, in convalescent depôts and other such places in Greece, and asked if his Department could not see their way to the utilisation of this skill to greater advantage by transferring it to France or some other active Front. Mr. Macpherson explained that the General Officer Commanding is aware of the position of all officers, including those possessing special qualifications, and that eminent doctors are at certain times employed in convalescent hospitals, being also at the nearest available spot to where there may be serious casualties.

